# Impression Management and Career Related Outcomes: A Systematic Literature Review

**DOI:** 10.3389/fpsyg.2021.701694

**Published:** 2021-07-30

**Authors:** Esraa Al-Shatti, Marc Ohana

**Affiliations:** ^1^Kedge Business School, Talence, France; ^2^Université de Pau et des Pays de l'Adour, Pau, France

**Keywords:** impression management, career, online, face to face, review–systematic, social media

## Abstract

Despite the popularity of the term impression management (IM) in the literature, there is no consensus as how different types of IM (direct vs. indirect) and modes of interaction (face-to-face vs. online) promote career-related outcomes. While most empirical studies focus on direct IM, individuals engage in both types of IM and interaction modes, particularly indirect IM in the online context. Indeed, recent developments suggest that online interactions now prevail over face-to-face interactions, especially during the COVID-19 pandemic. Accordingly, this study presents the first systematic literature review that differentiates between types of IM (direct vs. indirect) and modes of interaction (face-to-face vs. online) in a career development perspective. The review shows that direct IM is more widely studied in the face-to-face than online interaction mode, while indirect IM is neglected in both interaction modes. This study thus provides evidence of the need to investigate and differentiate between the different types of IM and interaction modes for career-related outcomes, highlighting some research gaps and directions for future inquiry.

## Introduction

In recent years, impression management (IM) has received renewed attention among scholars (e.g., Liu et al., 2019; Yang et al., 2021). IM can be defined as the process by which “individuals attempt to control the impressions others form of them” (Leary and Kowalski, 1990). IM is of primary importance for individuals, since the impressions they make on others influences how others perceive and treat them (Bozeman and Kacmar, 1997; Gioaba and Krings, 2017). Amongst other tools, IM has revolutionized career development, offering competitive and sustainable career opportunities (Villeda and McCamey, 2019). For example, developing a resume and showing daily achievements online are unavoidable tools to enhance our career (El Ouirdi et al., 2015). In this vein, individuals using IM have higher chances of getting positive job interview ratings (Amaral et al., 2019). A better understanding of the mechanism linking IM and the career-related outcomes requires distinguishing between different IM types, as different IM mechanisms might lead to different career outcomes.

First, IM may depend on the mechanism used, creating favorable impressions through two different types: direct and indirect IM. Direct IM refers to “individuals self-promoting their own achievements and success” (Tal-Or and Drukman, 2010). Indirect IM (also called impression management by association) refers to “behaviors undertaken by individuals at work through associations with other colleagues to create favorable impressions of themselves” (Cialdini and Richardson, 1980). Whereas, the literature mainly considers direct IM, indirect IM is now widely used, especially on social media platforms that are invading our lives. Using posts associated with a particular company/institute and connecting and following people on social network platforms are good examples of indirect IM. Unfortunately, evidence is lacking on the difference that direct and indirect IM might have on career outcomes.

Second, IM may depend on the interaction mode adopted: face-to-face or online (Zhao et al., 2008). Face-to-face interaction refers to the visibility of a physical body in social interactions, such as physical characteristics (i.e., gender, race, and looks), physical settings (i.e., furniture and decor), and personal attributes (i.e., appearance, language, and manner). Online interactions instead denote the invisibility of the physical body in social interactions through text or voice messages (Zhao et al., 2008). Very few studies explore the notion of IM in the online context. Since recruiters increasingly use social networking platforms in their search for candidates (Villeda and McCamey, 2019), understanding online IM for potential career consequences, and differentiating between the online and face-to-face contexts that lead to different career-related outcomes, is pivotal.

In this systematic literature review composed of 55 articles in English published from 1980 to 2020, we explore how the different IM mechanisms (i.e., direct vs. indirect, and face-to-face vs. online) contribute to individuals' career development, and seek to answer the following questions:

1. Are there any difference between IM types (direct vs. indirect) and career related outcomes?
2. Does IM have similar effects on career development in the face-to-face and online contexts?

A key contribution of this study is providing insights on the state-of-the-art of IM and the difference between the types (direct vs. indirect) and interaction modes (face-to-face vs. online) for a better understanding of the relevance of IM and the resulting career-related outcomes.

## Practical Insights Into the Prevalence of New Forms of IM

Since the COVID-19 pandemic, individuals and organizations have been forced to operate through online technologies and social platforms (Bhaskar et al., 2021). Consulting the social networking profiles of potential candidates on Twitter, Facebook, and LinkedIn is more than ever a fundamental human resource management practice in the modern organization (Villeda and McCamey, 2019). The information provided allows gauging the personality and interests of candidates and their alignment with the organizational culture.

The structure of social media enables individuals to share their achievements directly (direct IM) or through association with others (indirect IM). While direct IM has been widely examined (e.g., Andrews and Kacmar, 2001), indirect IM has become more salient in the contemporary context. Indeed, in the individual perspective, the extensive use of social media creates additional opportunities for indirect IM through allowing people to easily associate themselves with others on different social media platforms. In the organizational perspective, contemporary managers systematically consult social media that influence their professional decisions (Fieseler and Ranzini, 2015). For example, managers use social media to assess the suitability of a job seeker for a particular position (Van Iddekinge et al., 2016). Researching a job seeker's social media presence allows managers to see what others are saying about them. For instance, platforms such as LinkedIn allow users to recommend each other (considered indirect IM), and the testimonials on a user's platform can reveal what they might offer the company. Indirect IM is thus becoming fundamental in determining career outcomes.

As the prevalence and popularity of online social networking has grown extensively in recent years (Schivinski et al., 2020), IM has moved from the face-to-face to the online interaction mode. From the employee perspective, online social networking provides valuable resources, such as building business relations, identifying opportunities, and interacting with others (Nazir et al., 2020). From an organizational perspective, online social networking is recognized as a dominant communications tool (Dwivedi et al., 2020) that allows reducing recruitment costs (Leader-Chivée and Cowan, 2008). Interestingly, HR managers consider individuals' information on online social networking platforms as “honest” and accurate in comparison to the traditional résumé used in the face-to-face context (Zide et al., 2014). Likewise, Rowell (2010) shows that 70% of HR managers reject job applicants due to their online social networking behavior. The emergence and anchoring of new forms of IM lead us to differentiate between direct vs. indirect, and online vs. face-to-face IM, to understand their consequences on career outcomes.

## Theoretical Impression Management Perspective

Individuals manage their impressions for career path purposes as IM can enhance their career opportunities. We next present the different types of IM linked to career-related outcomes.

### Impression Management: From Direct to Indirect

Impression management refers to human behavior designed to obtain a favorable reaction from others (Felson, 1978; Bolino et al., 2008) through self-presentation (Goffman, 1959). IM theory was first conceptualized by Goffman (1959) who proposed a dramaturgical model of social life composed of two key players: an “actor” who engages in “IM tactics” and an “audience” that interacts with “actors” to create a desired image. IM tactics can be categorized as direct and indirect (Cialdini and Richardson, 1980). First, direct IM refers to individuals presenting their own achievements and success (Tal-Or and Drukman, 2010), including assertive and defensive tactics (Wayne and Kacmar, 1991; Stevens and Kristof, 1995). Assertive tactics are “proactive behaviors undertaken by individuals to create a specific identity to further their careers.” Defensive tactics are “reactive behaviors used by individuals following actions that may portray them negatively” seeking to “avoid negative career implications” (Andrews and Kacmar, 2001). Direct IM is premised to be linked with outcomes, including interview performance, job offers, hiring decisions, perceived qualifications, adequacy of information, and interviewer confidence (Gilmore and Ferris, 1989; Leary and Kowalski, 1990; Bolino et al., 2008).

Indirect IM refers to individuals managing their association with others for the purposes of creating a favorable impression of themselves (Cialdini and Richardson, 1980). Indirect tactics create impressions by involving a third party to manage the individual's image. Indirect IM supports the balance theory of Heider (1958) postulating that people tend to see things alike when they are associated with one another in order to maintain cognitive balance. According to Andrews and Kacmar (2001), indirect IM involves four connection-focused tactics: boasting, blurring, blaring, and burying. Boasting is defined as an individual embracing his or her positive connections by associating with favorable others. Burying is the individual tendency to conceal relationships with unsuccessful others for the sake of creating a perfect image of him or herself. Blaring is defined as an individual minimizing a connection with unfavorable others, especially in public. Finally, blurring refers to an individual's tendency to use the success of others, especially in the work place, as this will increase the perception of how successful he/she is in his/her career. Early work on indirect IM deems that it positively influences career-related outcomes, such as self-promotion (Cialdini, 1989).

### Impression Management: From Face-to-Face to Online

Whatever the tactics, IM is used in two interaction modes: face-to-face and online. While IM research is extensive, studies linking the phenomenon with online social networking and career-related outcomes are scarce. However, the fluidity of social media platforms, especially their ability to address multiple audiences and diverse purposes, renders the online context interesting to understand IM (Kaplan and Haenlein, 2010). Indeed, IM theory has been extended to the online context (Zhao et al., 2008; Hogan, 2010; Rosenberg and Egbert, 2011; Harrison and Budworth, 2015). Several researchers recognize the potential of online social networking and its relation to impression formation (Tong et al., 2008; Zhao et al., 2008). IM theory provides a framework to assess online impressions created by job seekers through the information they display (Barrick et al., 2009; Harrison and Budworth, 2015). Therefore, social media users ensure that their profile is catchy, aiming to influence how others perceive them (Rosenberg and Egbert, 2011). Indeed, individuals tend to follow and connect with particular people, companies, and associations for the sake of enhancing their image via indirect IM in the online context. This favorable image thus fosters positive career outcomes (El Ouirdi et al., 2015).

## The Systematic Literature Review Methodology

To explore the effects of the different IM tactics on career related outcomes, we conducted a systematic literature review (SLR). SLR involves gathering extant literature on a subject that meets the predetermined inclusion criteria and answers the established research question(s). Its purpose is to formulate a broad perspective of a research area and provide an unbiased summary of the literature (Torraco, 2005; Borrego et al., 2014). Moreover, a well-structured SLR has numerous benefits, such as explaining a specific problem, revealing gaps and inconsistencies in the literature, and providing guidance for future research and practice (Baumeister and Leary, 1997). The methodology also ensures the generation of knowledge in a structured and systematic way from multiple studies. One of the key advantages of SLR is that it allows the restrictive retrieval of data from multiple databases, ensuring it is comparatively less biased than traditional literature reviews (Borrego et al., 2014).

**Figure 1:**
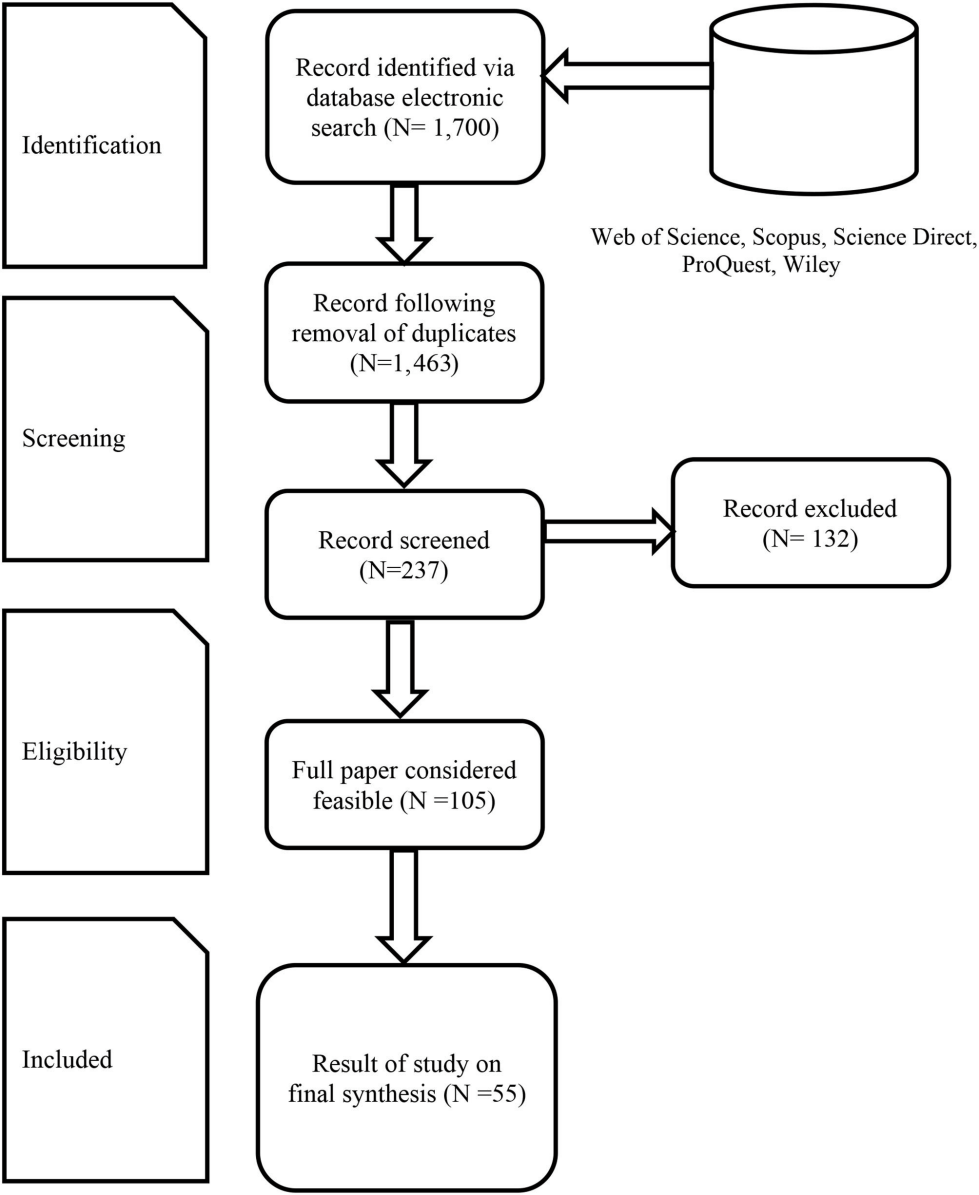
PRISMA flow diagram.

Different authors have presented guiding principles to assist SLR researchers in constructing procedures that adhere to the methodology and the strategies to evaluate suitable research (Nightingale, 2009). Following the four stages of Tranfield et al. (2003) in this review, we first defined the search strategy and identified potential databases before embarking on the search (Higgins and Green, 2008). Second, we identified suitable articles based on the predetermined inclusion and exclusion criteria. Third, we undertook a synthesis of the selected studies that involved extracting and categorizing the data. Last, we analyzed the results and drew conclusions. For the sake of transparency and to ensure our literature review is reproducible, all the relevant steps are detailed next. Figure 1 represents the Preferred Reporting Items for Systematic Reviews and Meta-Analyses (PRISMA) flow diagram (Moher et al., 2009). It allows to have a better overview of the different steps taken for this SLR.

### Searching the Empirical Literature

For this review, we checked different databases according to the IM definition. We then drew on seminal IM research papers to define the key terms and exact concepts later used to define the search terms and the time period. Based on these results and the information on the different terms used to describe IM, we identified the most promising search terms for our literature review as shown in Table 1 (e.g., impression management) using reliable databases, including Web of Science, Scopus, Science Direct, ProQuest, and Wiley. Web of Science and Scopus are citation databases that search multiple databases and sources to identify studies based on keywords, while the ProQuest search encompassed 18 databases.

**Table 1:** Summary of search result.

**No**.	**Keywords**	**WS**	**SC**	**SD**	**PQ**	**WI**
1	Impression Management	706	748	144	847	105
2	Impression Management + Human Resource	1	1	0	2	1
3	Impression Management + Career	6	5	0	11	1
4	Impression Management + Social Media	8	12	2	6	2
5	Impression Management + Social Networking	16	4	3	4	2
6	Impression Management + Online	11	16	2	10	3
7	Impression Management + by association	1	2	1	1	2
8	Impression Management + face-to-face	0	0	0	0	0
9	Impression Management + Job	24	28	1	32	8
10	Impression Management + connection focus	0	0	0	0	0
11	Impression Management + tactics	40	46	3	46	11
12	Impression Management + indirect	2	2	1	1	0
13	Impression Management + self-presentation	19	15	3	21	4
14	Impression Management + self-promotion	1	1	0	2	0

*WS, Web of Science; SC, Scopus; SD, Science Direct; PQ, ProQuest; WI, Wiley. The search results are limited to the title field*.

We used the following keywords in our search: *IM, HR, career, social media, social networking, online, impression management by association, face-to-face*, and *job search* in combination with *impression management*. We chose to begin our SLR in 1980 because critical theoretical IM frameworks were published at that time (Cialdini and Richardson, 1980).

After defining the research parameters, we performed the literature search initially resulting in 1,700 publications which we recorded and organized using Refworks and Excel (Callahan, 2010).

### Screening the Literature

We screened the resulting 1,700 publications in two steps. In the first step, we searched for relevant publications using the defined search terms and filtered the results for the related literature. Following the literature search recommendations (Brocke et al., 2015), we performed a backward (screening the references using these papers) and forward search (publications that cite these papers). Additionally, we followed Levy and Ellis's (2006) ranking approach to select the appropriate journals. For our selection process, we picked the top tier IM journals enriched with those that focus on similar or adjacent topics, as our study includes online social networking as well as individuals' career-related outcomes. We selected numerous reputable journals, including *Journal of Applied Social Psychology, Journal of Organizational Behavior, Academy of Management, Journal of Management, Journal of Computer-Mediated Communication, Career Development International*, amongst many more. After screening the literature, 237 articles remained.

### Inclusion and Exclusion Criteria

The inclusion and exclusion criteria were set as part of the protocol prior to the start of the project. Included manuscripts needed to be (a) academic-peer-reviewed, (b) the focus of these publication needed to be centered around impression management, (c) these papers needed to be specifically aligned with career related outcomes from different mode of interactions (i.e., face-to-face and online) (d) in English, (e) the year of publication had to fall between 1980 and 2020. In contrast, we excluded: (1) Publication in non-English format, (2) duplicated research papers, (3) non-peer-reviewed articles (such as non-academic journals), (3) unpublished doctoral theses, and (4) gray literature (such as conferences and working papers).

We considered the 237 articles for inclusion and exclusion. The first step in this process entailed removing unrelated papers, reducing the number of records to 105. Next, we checked the articles for their relevance to our study based on the title, keywords, and abstract. After evaluating all publications, we identified 55 articles as relevant to our research topic. Table 2 provides the authors/date and career related outcomes according to the direct and indirect IM, face-to-face and online research focus. We extracted those variables from the individual papers: salary, promotion, performance assessment, job promotion, job interview ratings, interviewers' judgements, job interview evaluation, hiring recommendations, job offer, second job interview, recruiter evaluation, promotion scores, performance appraisal, supervisor liking, performance ratings, career success, performance evaluation, salary progression, promotability assessments, influence job search, recruitment process, selection process, job interview assessment, job performance, job design, employee selection, job satisfaction, job commitment, career satisfaction, adjusted salary, self-promotion, salary recommendations, and job opportunities.

**Table 2:** Search results and classification.

	**Author/Date**	**Career-related outcomes**
Face-to-face direct IM	Higgins et al. (2003)	Salary, promotion, performance assessment
	Bolino et al. (2008)	Job promotion
	Baron (1993)	Job interview ratings (interview selection)
	Gilmore and Ferris (1989)	Influence interviewers' judgements
	Ellis et al. (2002)	Job Interview evaluation
	Higgins and Judge (2004)	Hiring recommendations, job offer
	Stevens and Kristof (1995)	Second job interview, job offer
	Kristof-Brown et al. (2002)	Job interview
	McFarland et al. (2003)	Recruiter evaluations, promotion scores
	Bolino and Turnley (2003)	Performance appraisal
	Wayne and Ferris (1990)	Supervisor liking, performance rating
	Judge and Bretz (1994)	Career success
	Ferris et al. (1994)	Performance evaluation
	Harris et al. (2007)	Performance ratings
	Treadway et al. (2007)	Performance ratings
	Wayne and Kacmar (1991)	Performance appraisal
	Wayne and Liden (1995)	Performance appraisal
	Barsness et al. (2005)	Performance appraisal
	Wayne et al. (1997)	Career success: performance ratings, salary progression, promotability assessments,
	Kacmar and Carlson (1994)	Influence job search and the recruitment process
	Swann et al. (2015)	Selection process
	Roulin and Bourdage (2017)	Job interview
	Gioaba and Krings (2017)	Job interview Job offer
	Von Baeyer et al. (1981)	Job interview
	Noor et al. (2017)	Job interview assessment
	Tsai et al. (2005)	Job interview evaluation
	Weiss and Feldman (2006)	Job interview
	Bourdage et al. (2017)	Job interview
	Tsai et al. (2010)	Job interview
	Chen et al. (2010)	Job interview
	Viswesvaran et al. (2001)	Job performance
	Zivnuska et al. (2004)	Job performance
	Foldes et al. (2006)	Job performance
	O'Connell et al. (2011)	Job performance
	Ispas et al. (2014)	Job performance
	Ingold et al. (2015)	Job performance
	Brouer et al. (2016)	Job performance
	Peck and Levashina (2017)	Job interview, job performance
	Probst et al. (2019)	Job performance
	Kacmar and Carlson (1999)	Job interview, performance appraisal
	Law et al. (2002)	Job offer
	Westphal (2010)	Job design
	Avery and McKay (2006)	Employee selection
	Harris et al. (2013)	Job satisfaction
	Asawo and George (2018)	Job commitment
	Cheng et al. (2014)	Career success: job performance, career satisfaction, adjusted salary
Face-to-face indirect IM	Cialdini and Richardson (1980)	Self-promotion
	Cialdini and de Nicholas (1989)	Self-promotion
	Finch and Cialdini (1989)	Self-promotion
	Andrews and Kacmar (2001)	Developed connection-focused tactics scale
Online direct IM	Rosenberg and Egbert (2011)	Self-promotion
	Stopfer et al. (2013)	Self-promotion
	Nestler and Back (2013)	Self-promotion
	Harrison and Budworth (2015)	Hiring and salary recommendations
	Paliszkiewicz and Madra-Sawicka (2016)	Job opportunities
Online indirect IM	No studies found	No studies found

### Data Management and Analysis

To identify the current and future research topics according to the 55 articles identified, we developed a framework to classify the articles. As a starting point, we analyzed studies in the face-to-face context, including career-related outcomes of direct and indirect IM. Then, we extended our analysis to the online context. Table 3 shows the classification by frequency, differentiating between the interaction modes (face-to-face vs. online) and career-related outcomes of the IM mechanisms (direct vs. indirect) adopted. Separating the interaction modes (face-to-face vs. online) allows identifying the gap in the literature and illustrating the importance of understanding both modes and IM tactics to achieve the desired career-related outcome.

**Table 3:** IM studies.

	**Type**	**Frequency**	**Percentage**
IM study focus	Face-to-face direct IM	46	79%
	Face-to-face indirect IM	4	12%
	Online direct IM	5	9%
	Online indirect IM	0	0%
	Total	55	100%

Indeed, while most of the studies identified deal with direct IM in the online context, some older studies explore the link between indirect face-to-face IM and career-related outcomes. However, while several authors integrate direct IM in the online context, we found no studies dealing with indirect IM in the online context.

## Findings

### Direct IM in the Face-to-Face Context

Regarding direct IM in the face-to-face context, most studies focus on the link between IM and job interview, job performance, and other career-related outcomes as detailed next.

#### Direct Face-to-Face IM and Job Interview

Numerous studies focus on the effect of direct face-to-face IM, with job interview as the most common career-related outcome (Gilmore and Ferris, 1989; Baron, 1993; Ellis et al., 2002; Kristof-Brown et al., 2002; Law et al., 2002; Weiss and Feldman, 2006; Noor et al., 2017; Peck and Levashina, 2017; Roulin and Bourdage, 2017). For instance, Von Baeyer et al. (1981) study a male interviewer's knowledge and attitude toward female candidates in a stereotyped environment. Kacmar and Carlson (1994) focus on the process of women searching for jobs using direct IM. Stevens and Kristof (1995) examine the relationship between direct IM and job interview outcome. Tsai et al. (2005) explore the effect of direct IM tactics on job interviews, showing these have a positive influence on interviewer evaluation. Chen et al. (2010) study applicant direct IM tactics in job interviews with the moderating role of interviewer affectivity. Empirical evidence shows that direct IM tactics, such as self-focused IM, other-focused IM, and non-verbal IM, positively influence interviewer evaluations through self-focused direct IM.

Tsai et al. (2010) study direct IM tactics in job interviews with an emphasis on three defensive applicant tactics: apologies, justifications, and excuses. Collecting empirical data through observing applicant interviews, they explore the moderating effect of negative competence- and integrity-related concerns on the three direct IM defensive tactics, finding that the apologies tactic has the strongest impact.

Swann et al. (2015) study direct IM and job interviews in the medical context. Although unable to provide conclusive evidence, the authors offer a brief overview of direct IM over time, and encourage training models that provide a logical and systematic approach for candidates to ensure that the results of interview selection are closely correlated with good clinical outcomes for successful candidates. Bourdage et al. (2017) show the difference between reality and faking in job interviews. Direct IM is used to impress interviewers, as candidates attempt to create a likable impression and gain job opportunities. They approach direct IM from various perspectives, such as being honest and deceptive, IM effectiveness, IM as a shield against discrimination, and IM as dyadic and beyond the applicant.

Gioaba and Krings (2017) study effective ways of mitigating discrimination against older applicants based on direct IM in job interviews. They find that the use of direct IM by older applicants provides stronger job interview and hiring opportunities. Similarly to Bourdage et al. (2017), Roulin and Bourdage (2017) extend the study of the use of honesty and deceptive direct IM across multiple job interviews.

Overall, these studies show that direct IM tactics lead to positive effects on job interviews in the face-to-face interaction mode.

#### Direct Face-to-Face IM and Job Performance

Numerous scholars study the positive effect of direct IM in the face-to-face interaction mode on individuals' job performance (Wayne and Ferris, 1990; Wayne and Kacmar, 1991; Ferris et al., 1994; Wayne and Liden, 1995; Bolino and Turnley, 2003; Zivnuska et al., 2004; Barsness et al., 2005; Foldes et al., 2006; Harris et al., 2007; O'Connell et al., 2011).

Viswesvaran et al. (2001) study direct face-to-face IM and job performance by exploring the relationship between direct IM scale scores, overall job performance, and managerial interpersonal interactions. Zivnuska et al. (2004) investigate the interactive effect of organizational politics and direct IM on supervisor ratings of employee performance. In their study, Ispas et al. (2014) find a significant link between direct IM and objective job performance. Another study in the field of direct IM and job performance is that of Ingold et al. (2015) who focus on direct IM, faking in the selection context, and job performance. The authors find that candidates that faked direct IM in interviews also falsified a personality inventory, and that this deceit is positively related to supervisor job performance rating.

Brouer et al. (2016) study direct IM and the ability to manage resources with job performance as mediator. They find that higher levels of social resources, such as reputation and leader-member exchange, are positively related to job performance. Peck and Levashina (2017) study direct IM in relation to interviews and job performance, finding that direct IM has a stronger impact on interview and job performance rating. The most recent study is that of Probst et al. (2019) investigating the relationship between job insecurity and direct IM to determine a relationship between supervisor-focused IM, lower job insecurity, positive in-role behavior, and job performance. Accordingly, if direct IM is correctly practiced, irrespective of whether true or false, it will lead to a better job performance rating.

Overall, the use of direct IM at the workplace has a positive effect on employees' job performance rating.

#### Direct Face-to-Face IM and Other Career Outcomes

Several studies link direct face-to-face IM and different career-related outcomes, such as salary increase, hiring recommendations, promotions, job commitment, and overall career success (Judge and Bretz, 1994; Kacmar and Carlson, 1994; Wayne et al., 1997; Higgins et al., 2003; McFarland et al., 2003; Higgins and Judge, 2004; Avery and McKay, 2006; Bolino et al., 2008; Westphal, 2010; Asawo and George, 2018).

For example, Kang et al. (2012) investigate the relationship between job insecurity and IM work-related behaviors, finding that the perception of job insecurity leads to reduced extra-role and IM behavior. Evidently, the intensity of withdrawal increases with increased employability. Harris et al. (2013) study IM behaviors in relation to IM culture and job outcomes, such as performance, promotion, compensation, and IM tactics (intimidation and exemplification), finding that intimidation has negative effects, while exemplification has positive effects on IM tactics. Cheng et al. (2014) focus on the interactive effects of task performance and IM tactics on career outcomes, finding that the relationship between task performance and career satisfaction is greater among employees who frequently use self-promotion.

Generally, in the face-to-face interaction mode, a strong relation is found between direct IM and career-related outcomes, such as salary increase, job promotion, job commitment, and hiring recommendations.

### Indirect IM in the Face-to-Face Context

While generally few scholars focus on indirect compared to direct IM, some studies consider indirect IM in the face-to-face interaction mode with different career-related outcomes, such as job engagement and job satisfaction. Cialdini and Richardson (1980) show that individuals tend to use indirect IM tactics, for example, highlighting successful connections with others to enhance their personal image (prestige). Cialdini (1989) shows that an individual's image can be enhanced by associating with successful others and disassociating from failures. Finch and Cialdini (1989) reveal that unit-connection plays an essential role in individuals' image simply by associating themselves by birth date with favorable or unfavorable individuals. Finally, Andrews and Kacmar (2001) develop and validate an indirect IM scale, albeit not adopted in relation to career-related outcomes.

Overall, these studies show that indirect IM has positive effects on career-related outcomes that are underestimated.

### Direct IM in the Online Context

Some researchers have recently focused on direct IM in the online context (Rosenberg and Egbert, 2011; Nestler and Back, 2013; Stopfer et al., 2013; Harrison and Budworth, 2015; Paliszkiewicz and Madra-Sawicka, 2016). For instance, Paliszkiewicz and Madra-Sawicka (2016) illustrate the importance of online IM on LinkedIn to benefit from the platform features and gain job opportunities. Harrison and Budworth (2015) find a positive relation between verbal and non-verbal IM on hiring and salary recommendations in social media platforms. Three studies deal with the importance of IM in online social networks for self-promotion purposes (Rosenberg and Egbert, 2011; Nestler and Back, 2013; Stopfer et al., 2013).

Overall, these studies show the importance of using direct IM in the online context to obtain the desired career outcomes.

### Indirect IM in the Online Context

As mentioned, we found no studies that deal specifically with indirect IM in the online context.

## Discussion

**Figure 2:**
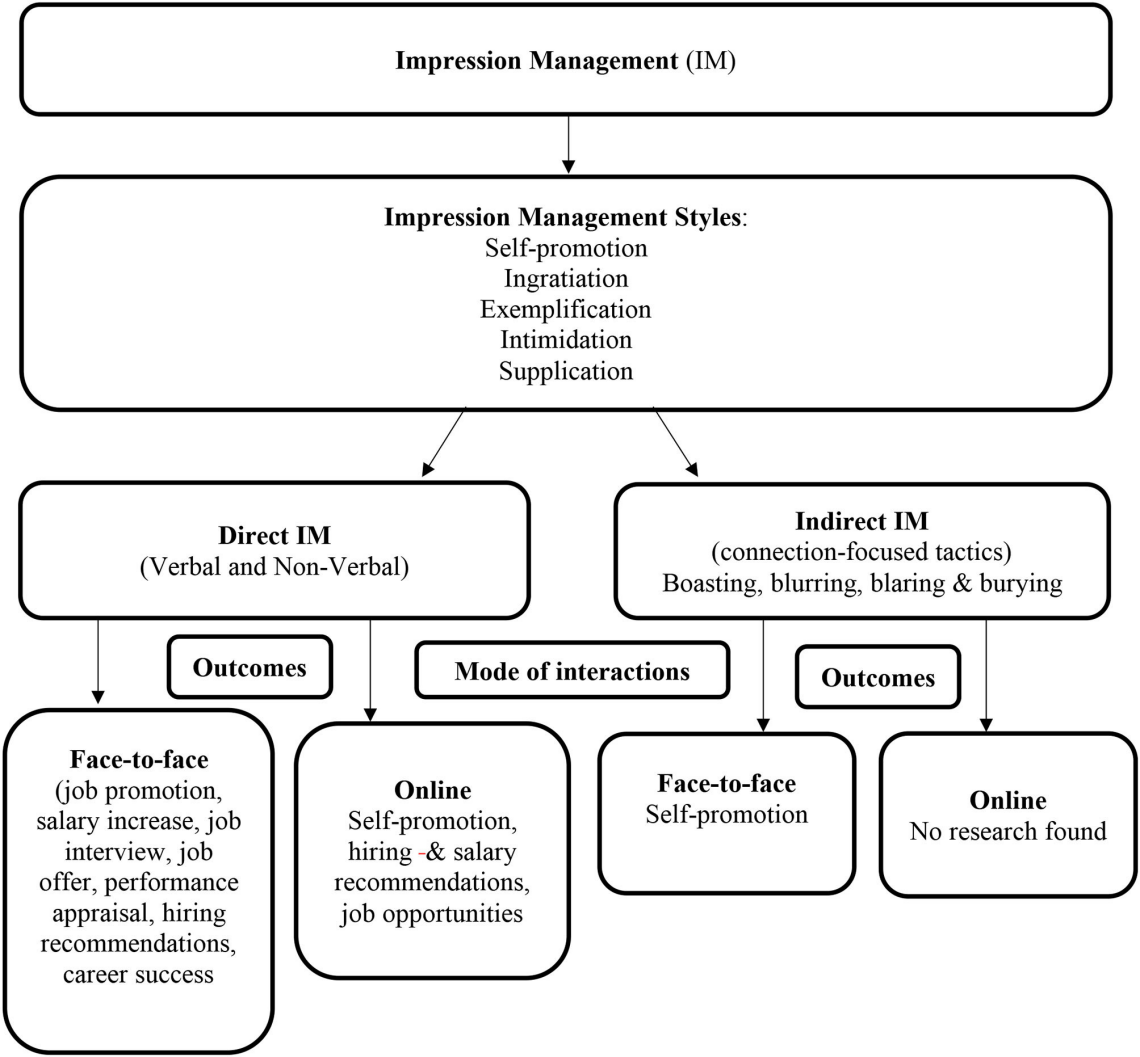
IM scheme.

Our analysis of the 55 studies provides deep insights on IM and career-related outcomes in both the face-to-face and online context. Figure 2 provides an illustration of what is IM and what are its associations.

Individuals shape their impressions in accordance with how they represent themselves and what they want to show recruiters and managers, both in a direct and indirect way, and in the face-to-face and online contexts. We do not observe any major differences between IM mechanisms (direct vs. indirect) and interaction mode (face-to-face vs. online) in relation to their positive role in career-related outcomes.

Indeed, both the direct vs. indirect IM mechanisms are linked to individuals' career success. Regarding career-related outcomes, such as job interviews and job performance, most studies naturally relate to direct IM in the face-to-face interaction mode. In the very few studies that deal with the online interaction mode, self-promotion is the common career-related outcome. For a better overview of IM in all contexts and circumstances, further research is needed on the different potential outcomes. For example, in the online mode, it may be worth exploring whether IM is so powerful that it impacts job performance despite the absence of direct physical interactions.

In addition, scholars have focused mainly on direct IM, neglecting the importance of indirect IM. In particular, no studies focus on the indirect online interaction mode, while only 4 deal with indirect face-to-face interactions. There is thus a gap in the literature in differentiating between direct vs. indirect IM in the face-to-face vs. online interaction modes, which is crucial to career-related outcomes. To fully capture the IM phenomenon, scholars should consider the impact of indirect IM in general, and specifically in the online context, on career-related outcomes.

Our review clearly shows the need to differentiate IM (direct vs. indirect) in both contexts (face-to-face vs. online) and the career-related outcomes. Even if not our main aim, this systematic literature review allows highlighting some additional unanswered questions for future researchers to address as specified in Table 4.

**Table 4:** Indirect IM questions and future research avenues.

**What is indirect IM?**
Unintended indirect IM in the job search context
Effects of indirect IM in the job search context: online vs. face-to-face
Deceptive vs. honest indirect IM in the job search context
**What indirect IM behaviors have been identified?**
Which connection-focused tactic is mostly used in the job search context?
Combination or single use of connection-focused tactics?
**What motivates individuals to manage their indirect IM?**
Antecedents of indirect IM
Indirect IM in the online and face-to-face context
Unintended use of indirect IM in the job search context
Intended use of indirect IM in the job search context
**Are some individuals better at indirect IM than others?**
Influence of social networking platforms on individuals' indirect IM
Job seekers use of indirect IM vs. employers' reactions to indirect IM
**What are the social networking implications of indirect IM?**
Is building relationships online the main factor of indirect IM?
How do individuals using social networking react to indirect IM?

### Implications for Individuals and Career Counselors

Research on IM (direct vs. indirect) has practical implication for individuals and career counselors. For individuals, making the right association with successful others and disassociating from unsuccessful others is a significant element in succeeding at work and enhancing prestige (Andrews and Kacmar, 2001). Further, the literature shows that individuals who create an impression need to maintain this impression even at later stages to manage and strengthen the image in the minds of others (Higgins et al., 2003; Barrick et al., 2009). First, associating with a third party is theoretically proven to create a cognitive balance in the mind of the others (Kacmar et al., 2011). As such, individuals in the workplace engage with higher reputation individuals and learn from the best because associating with unfavorable others will affect their career outcome. Second, individuals using online social networking must pay attention to who they are connected and associated with, as this will lead to either valuable or adverse future returns. Individuals are frequently evaluated for career purposes enabled by the accessibility of social network platforms.

A better understanding of online social networking is also crucial for career counselors to stay up to date with digital trends. Scholars indicate the emergence of online social networking for both job seekers and career counselors (Bolino et al., 2016). According to the analysis of social networking platforms, career counselors mainly use LinkedIn when checking individuals or job seekers for career purposes, as it is used more for professional networking, while Facebook and Twitter also share non-professional content, potentially leading to bias. However, every jobseeker has a social life, and rejecting individuals because of their Facebook content may lead to disregarding those who could in fact benefit the organization.

### Limitations

This SLR took great care to avoid any publication bias. First, the Preferred Reporting Items in Systematic Reviews and Meta-Analysis (PRISMA) flow diagram shows the clarity and credibility of our research (Moher et al., 2009). This universally accepted evidence-based checklist reduces publication bias. Second, we were highly concerned about the gray literature. However, we decided to remove gray literature from our inclusion criteria. Gray literature is composed of working papers, conferences and articles that are not academically peer-reviewed (Adams et al., 2016). We are aware that some authors encourage to include gray literature (e.g., Briner and Denyer, 2012). However, we follow the recommendation of Kraus et al. (2020) to exclude it. Traditional reviews are criticized for subjective literature selection and quality appraisal (Denyer and Tranfield, 2006). Indeed, by integrated peer review articles, the process is more transparent and replicable. Also, the selected papers have been checked through the academic process. It thus represents a guarantee of quality. We acknowledge that this strategy can still be responsible of a publication bias as all papers of good quality are not all published in peer reviewed journals. Third, we considered five main and highly reliable database to reduce the publication bias such as Web of Science, Scopus, Wiley, Science Direct and Proquest. Finally, the included papers were checked by two authors to enhance the credibility and to evaluate the quality of the methodology of the papers that are included in the SLR. Because we chose only peer reviewed articles in main research database and because all papers have been checked by two authors to detect any quality problem, we can ensure a good methodological quality of the included studies.

Besides, our aim was to do a systematic literature review in order to compare direct vs. indirect and online vs. face to face IM. Unfortunately, due to the weak number of peer reviewed publications about indirect IM and online IM, a quantitative meta-analysis would have not been appropriated. Nevertheless, it would be very insightful to do in the future a quantitative analysis of the impact of different types of IM on career related outcomes when more publications will be available.

## Conclusion

This literature review shows that indirect IM is often overlooked by researchers, highlighting the need for further investigations on both interactions modes (face-to-face vs. online). While the literature shows that job seekers and recruiters use online social networking to create a positive image, the field has received limited academic attention, and further research is needed to understand this phenomenon in greater detail.

## Data Availability Statement

The original contributions presented in the study are included in the article/supplementary material, further inquiries can be directed to the corresponding author.

## Author Contributions

EA-S did the SLR. MO helped in the writing of the paper. All authors listed have made a substantial, direct and intellectual contribution to the work, and approved it for publication.

## Conflict of Interest

The authors declare that the research was conducted in the absence of any commercial or financial relationships that could be construed as a potential conflict of interest.

## Publisher's Note

All claims expressed in this article are solely those of the authors and do not necessarily represent those of their affiliated organizations, or those of the publisher, the editors and the reviewers. Any product that may be evaluated in this article, or claim that may be made by its manufacturer, is not guaranteed or endorsed by the publisher.
